# 4-(4-Chloro­phen­yl)-6-(methyl­sulfan­yl)pyrimidin-2-amine

**DOI:** 10.1107/S1600536809024891

**Published:** 2009-07-08

**Authors:** Qi-Hua Zhao, Li-Nan Li, Kun-Miao Wang

**Affiliations:** a School of Chemical Science and Technology, Key Laboratory of Medicinal Chemistry for Natural Resources, Ministry of Education, Yunnan University, Kunming 650091, People’s Republic of China

## Abstract

In the title compound, C_11_H_10_ClN_3_S, the dihedral angle between the benzene and pyrimidine rings is 3.99 (4)°. In the crystal, inter­molecular N—H⋯N hydrogen bonds link the mol­ecules into ribbons of *R* ^2^_2_(8) rings parallel to [100]. Weak C—H⋯S contacts connect adjacent ribbons into a two-dimensional undulating layer-like structure extending parallel to (110). The benzene and pyrimidine rings of adjacent mol­ecules have the offset face-to-face π–π stacking inter­actions in a zigzag fashion along the *c* axis, with perpendicular ring distances of 3.463 and 3.639 Å, and a dihedral angle between the planes of 3.99 (2)°. The distance between the ring centroids is 4.420 (2) Å.

## Related literature

For the synthesis of pyrimidine-5-carbaldehydes from α-formyl­aroylketene dithio­acetals, see: Mathews & Asokan (2007 ▶). For the synthesis of a 6-aryl amino­pyrimidine compound, see: Lin *et al.* (2008 ▶). For the application of organic compounds as ligands, see: Li *et al.* (2007 ▶). For the importance amino­pyrimidine compounds in the synthesis of complexes, see: Cui & Lan (2007 ▶). For a review of inter­molecular C—H⋯S contacts, see: Taylor & Kennard (1982 ▶). For graph-set notation, see: Bernstein *et al.* (1995 ▶).
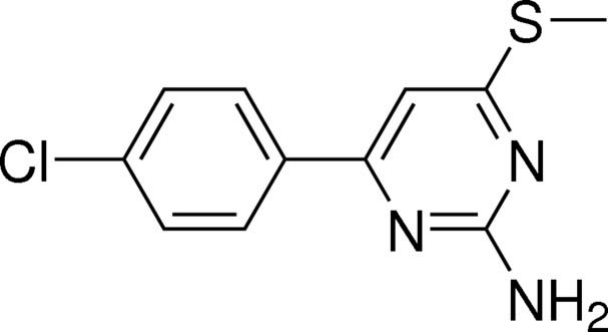


## Experimental

### 

#### Crystal data

C_11_H_10_ClN_3_S
*M* *_r_* = 251.73
Orthorhombic, 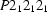

*a* = 6.8148 (11) Å
*b* = 10.6107 (16) Å
*c* = 16.509 (3) Å
*V* = 1193.7 (3) Å^3^
*Z* = 4
Mo *K*α radiation
μ = 0.47 mm^−1^
*T* = 293 K
0.25 × 0.14 × 0.08 mm

#### Data collection

Bruker APEXII 1K CCD area-detector diffractometer
Absorption correction: multi-scan (*SADABS*; Sheldrick, 1996 ▶) *T*_min_ = 0.925, *T*_max_ = 0.964
7825 measured reflections
2800 independent reflections
1841 reflections with *I* > 2σ(*I*)
*R*_int_ = 0.050

#### Refinement

*R*[*F* ^2^ > 2σ(*F* ^2^)] = 0.049
*wR*(*F* ^2^) = 0.096
*S* = 1.01
2800 reflections
145 parameters
H-atom parameters constrained
Δρ_max_ = 0.19 e Å^−3^
Δρ_min_ = −0.20 e Å^−3^
Absolute structure: Flack (1983 ▶), 1061 Friedel pairs
Flack parameter: 0.02 (10)

### 

Data collection: *APEX2* (Bruker, 2004 ▶); cell refinement: *SAINT* (Bruker, 2004 ▶); data reduction: *SAINT*; program(s) used to solve structure: *SHELXS97* (Sheldrick, 2008 ▶); program(s) used to refine structure: *SHELXL97* (Sheldrick, 2008 ▶); molecular graphics: *SHELXTL* (Sheldrick, 2008 ▶); software used to prepare material for publication: *SHELXTL* and *PLATON* (Spek, 2009 ▶).

## Supplementary Material

Crystal structure: contains datablocks I, global. DOI: 10.1107/S1600536809024891/si2183sup1.cif

Structure factors: contains datablocks I. DOI: 10.1107/S1600536809024891/si2183Isup2.hkl

Additional supplementary materials:  crystallographic information; 3D view; checkCIF report

## Figures and Tables

**Table 1:** Hydrogen-bond geometry (Å, °)

*D*—H⋯*A*	*D*—H	H⋯*A*	*D*⋯*A*	*D*—H⋯*A*
N3—H3*A*⋯N2^i^	0.86	2.31	3.095 (3)	152
N3—H3*B*⋯N1^ii^	0.86	2.21	3.045 (3)	164
C11—H11*A*⋯S1^iii^	0.96	2.93	3.859 (4)	163

Symmetry codes: (i) 
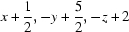
; (ii) 
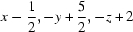
; (iii) 
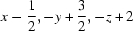
.

**Fig. 1.**
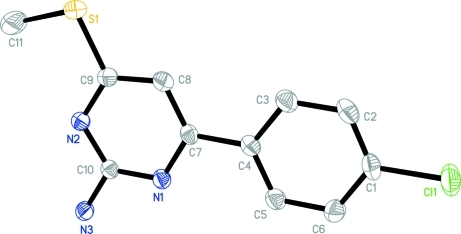
The molecular structure of the title compound with the atom-numbering scheme. Displacement ellipsoids are drawn at the 30% probability level.

**Fig. 2.**
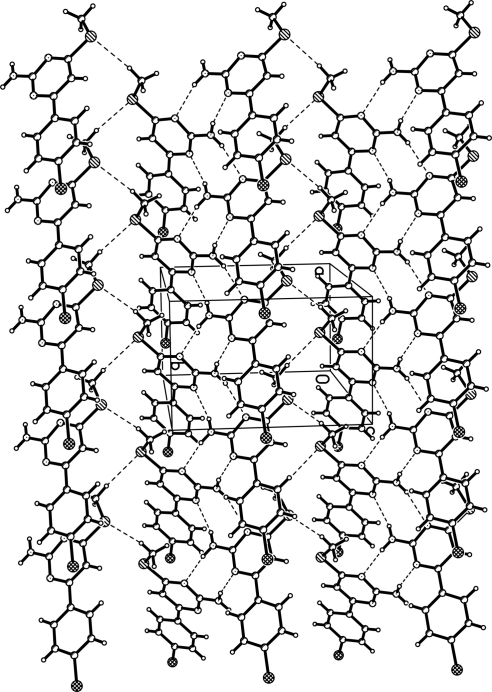
The crystal packing diagram of the title compound, showing hydrogen bonds along the ab plane.

## supplementary crystallographic information

### Comment

In recent years, aminopyrimidine compounds have shown predominant bioactivity and played an important role in the drug synthesis (Mathews & Asokan, 2007). Meanwhile, these organic compounds can be used as new organic N-donor ligands, which can construct a wide range of coordination polymers with novel architectures and special properties. The selection and synthesis of proper ligands are the most important tasks (Li *et al.*, 2007; Cui & Lan, 2007). Herein, the crystal structure of the title compound is reported. Its synthetic method followed the procedure given by Lin *et al.*, (2008).

In the title compound (Fig. 1), all atoms are almost in the same plane and the largest distortion from the mean plane being 0.3127Å for C11, atoms S1 and Cl1 being 0.1338 (6) and 0.1251 (5) Å out-of-plane. The two aromatic rings of the molecule make a dihedral angle of 3.99 (4)°.

In the crystal structure, there are two kinds of hydrogen bonds. One group is N3—H3A···N2, N3—H3B···N1, and the other is C11—H11A···S1 (Fig. 2 and Table 1). The strong intermolecular N—H···N hydrogen bonds link the molecules into ribbons of R^2^_2_(8) rings (Bernstein *et al.* 1995) parallel to [1 0 0]. The weak intermolecular C—H···S contacts connect the adjacent ribbons into a two-dimensional waved layer-like structure extending parallel to (1 1 0). Similar geometric parameters (H···S = 2.916 Å, angle C—H···S = 164<%) were discussed for a possible intermolecular C—H···S contact by Taylor & Kennard (1982). The phenyl and pyrimidine rings of adjacent molecules exhibit π—π stacking interactions in a zig-zag fashion along the *c* axis with perpendicular ring distances of 3.463 Å and 3.639 Å, and the dihedral angle α being 3.99°. The distance between the ring centroids *Cg1*···*Cg2^iv^* amounts to 4.420 (2) Å. Cg1 and Cg2 represent the centroids of the pyrimidine and phenyl rings. The symmetry code: (iv = -1 + *x*, *y*, *z*). The intermolecular forces construct a three-dimensional supramolecular architecture in the crystal.

### Experimental

All chemicals used were commercially available. We first got the title compound using the reported method (Lin *et al.*, 2008). After that 1 mmol (0.025 g) 4-(4-Chlorophenyl)-6-(methylthio)pyrimidin-2-amine was dissolved in a mixture of 15 ml methyl cyanide and 5 ml water. Then the solution was stirred for 40 min at room temperature. The solvent was removed gradually for a few weeks and faint yellow crystals for X-ray data collection were obtained by the slow evaporation method.

### Refinement

H atoms bonded to C and N atoms were calculated geometrically and allowed to ride on the C and N atoms with distance restraints of C—H = 0.93 Å and N—H = 0.86 Å, with *U*_iso_(H) = 1.2*U*_eq_(C, N).

### Crystal data

**Table:**

C_11_H_10_ClN_3_S	*F*(000) = 520
*M**_r_* = 251.73	*D*_x_ = 1.401 Mg m^−^^3^
Orthorhombic, *P*2_1_2_1_2_1_	Mo *K*α radiation, λ = 0.71073 Å
Hall symbol: P 2ac 2ab	Cell parameters from 3984 reflections
*a* = 6.8148 (11) Å	θ = 2.3–28.4°
*b* = 10.6107 (16) Å	µ = 0.47 mm^−^^1^
*c* = 16.509 (3) Å	*T* = 293 K
*V* = 1193.7 (3) Å^3^	Block, yellow
*Z* = 4	0.25 × 0.14 × 0.08 mm

### Data collection

**Table:**

Bruker APEXII 1K CCD area-detector diffractometer	2800 independent reflections
Radiation source: fine-focus sealed tube	1841 reflections with *I* > 2σ(*I*)
graphite	*R*_int_ = 0.050
φ and ω scans	θ_max_ = 28.4°, θ_min_ = 2.3°
Absorption correction: multi-scan (*SADABS*; Sheldrick, 1996)	*h* = −8→8
*T*_min_ = 0.925, *T*_max_ = 0.964	*k* = −13→11
7825 measured reflections	*l* = −21→22

### Refinement

**Table:**

Refinement on *F*^2^	Secondary atom site location: difference Fourier map
Least-squares matrix: full	Hydrogen site location: inferred from neighbouring sites
*R*[*F*^2^ > 2σ(*F*^2^)] = 0.049	H-atom parameters constrained
*wR*(*F*^2^) = 0.096	*w* = 1/[σ^2^(*F*_o_^2^) + (0.036*P*)^2^] where *P* = (*F*_o_^2^ + 2*F*_c_^2^)/3
*S* = 1.00	(Δ/σ)_max_ < 0.001
2800 reflections	Δρ_max_ = 0.19 e Å^−^^3^
145 parameters	Δρ_min_ = −0.20 e Å^−^^3^
0 restraints	Absolute structure: Flack (1983), 1061 Friedel pairs
Primary atom site location: structure-invariant direct methods	Flack parameter: 0.02 (10)

### Special details

**Table:**

Experimental. All e.s.d.'s (except the e.s.d. in the dihedral angle between two l.s. planes) are estimated using the full covariance matrix. The cell e.s.d.'s are taken into account individually in the estimation of e.s.d.'s in distances, angles and torsion angles; correlations between e.s.d.'s in cell parameters are only used when they are defined by crystal symmetry. An approximate (isotropic) treatment of cell e.s.d.'s is used for estimating e.s.d.'s involving l.s. planes.
Geometry. All e.s.d.'s (except the e.s.d. in the dihedral angle between two l.s. planes) are estimated using the full covariance matrix. The cell e.s.d.'s are taken into account individually in the estimation of e.s.d.'s in distances, angles and torsion angles; correlations between e.s.d.'s in cell parameters are only used when they are defined by crystal symmetry. An approximate (isotropic) treatment of cell e.s.d.'s is used for estimating e.s.d.'s involving l.s. planes.
Refinement. Refinement of *F*^2^ against ALL reflections. The weighted *R*-factor *wR* and goodness of fit *S* are based on *F*^2^, conventional *R*-factors *R* are based on *F*, with *F* set to zero for negative *F*^2^. The threshold expression of *F*^2^ > σ(*F*^2^) is used only for calculating *R*-factors(gt) *etc*. and is not relevant to the choice of reflections for refinement. *R*-factors based on *F*^2^ are statistically about twice as large as those based on *F*, and *R*- factors based on ALL data will be even larger.

### Fractional atomic coordinates and isotropic or equivalent isotropic displacement parameters (Å^2^)

**Table:**

	*x*	*y*	*z*	*U*_iso_*/*U*_eq_	
Cl1	1.15208 (14)	1.01534 (10)	0.62970 (5)	0.0694 (3)	
S1	−0.01688 (13)	0.85410 (8)	0.91781 (5)	0.0522 (2)	
N1	0.4426 (3)	1.1541 (2)	0.89640 (13)	0.0357 (6)	
N2	0.1442 (4)	1.0763 (2)	0.95722 (13)	0.0338 (6)	
N3	0.2824 (4)	1.2627 (2)	0.99649 (16)	0.0423 (6)	
H3A	0.3713	1.3201	0.9931	0.051*	
H3B	0.1880	1.2708	1.0307	0.051*	
C1	0.9466 (5)	1.0272 (3)	0.69138 (17)	0.0454 (8)	
C2	0.8052 (5)	0.9354 (3)	0.68825 (18)	0.0508 (9)	
H2B	0.8186	0.8682	0.6526	0.061*	
C3	0.6427 (5)	0.9430 (3)	0.73817 (19)	0.0473 (8)	
H3C	0.5474	0.8803	0.7359	0.057*	
C4	0.6197 (4)	1.0437 (3)	0.79209 (16)	0.0359 (7)	
C5	0.7638 (5)	1.1359 (3)	0.79272 (17)	0.0427 (8)	
H5A	0.7502	1.2044	0.8274	0.051*	
C6	0.9275 (5)	1.1292 (3)	0.74324 (18)	0.0484 (9)	
H6A	1.0228	1.1919	0.7448	0.058*	
C7	0.4499 (4)	1.0503 (3)	0.84876 (16)	0.0343 (7)	
C8	0.3107 (5)	0.9576 (3)	0.85466 (18)	0.0438 (8)	
H8A	0.3171	0.8859	0.8224	0.053*	
C9	0.1589 (4)	0.9737 (3)	0.91043 (17)	0.0366 (7)	
C10	0.2911 (4)	1.1611 (3)	0.94864 (16)	0.0333 (7)	
C11	−0.1656 (6)	0.9020 (4)	1.0015 (2)	0.0811 (13)	
H11A	−0.2674	0.8410	1.0102	0.122*	
H11B	−0.2235	0.9826	0.9900	0.122*	
H11C	−0.0858	0.9083	1.0493	0.122*	

### Atomic displacement parameters (Å^2^)

**Table:**

	*U*^11^	*U*^22^	*U*^33^	*U*^12^	*U*^13^	*U*^23^
Cl1	0.0647 (6)	0.0800 (7)	0.0634 (6)	0.0144 (6)	0.0276 (5)	−0.0046 (5)
S1	0.0488 (5)	0.0391 (4)	0.0686 (5)	−0.0083 (5)	0.0010 (5)	−0.0064 (5)
N1	0.0339 (13)	0.0370 (14)	0.0361 (13)	0.0013 (12)	0.0010 (10)	−0.0044 (12)
N2	0.0334 (13)	0.0344 (14)	0.0336 (13)	0.0003 (12)	−0.0039 (11)	−0.0010 (12)
N3	0.0381 (16)	0.0411 (16)	0.0476 (14)	−0.0054 (12)	0.0095 (13)	−0.0139 (13)
C1	0.048 (2)	0.053 (2)	0.0349 (16)	0.0154 (18)	0.0071 (15)	0.0003 (16)
C2	0.056 (2)	0.049 (2)	0.0468 (19)	0.0101 (18)	0.0025 (18)	−0.0163 (17)
C3	0.045 (2)	0.046 (2)	0.0516 (19)	0.0008 (17)	−0.0012 (17)	−0.0137 (17)
C4	0.0367 (18)	0.0368 (18)	0.0344 (15)	0.0061 (14)	−0.0056 (13)	−0.0035 (14)
C5	0.0501 (19)	0.0398 (19)	0.0381 (17)	0.0023 (18)	0.0065 (15)	−0.0044 (16)
C6	0.051 (2)	0.049 (2)	0.0446 (18)	−0.0049 (17)	0.0095 (16)	−0.0024 (17)
C7	0.0310 (17)	0.0373 (18)	0.0347 (15)	0.0058 (14)	−0.0033 (12)	−0.0044 (14)
C8	0.044 (2)	0.0382 (19)	0.0489 (19)	−0.0005 (16)	0.0015 (15)	−0.0105 (15)
C9	0.0346 (16)	0.0341 (17)	0.0411 (16)	0.0000 (14)	−0.0101 (15)	0.0027 (15)
C10	0.0319 (16)	0.0346 (18)	0.0334 (15)	0.0036 (14)	−0.0043 (12)	−0.0021 (14)
C11	0.077 (3)	0.065 (3)	0.101 (3)	−0.021 (2)	0.037 (3)	−0.001 (2)

### Geometric parameters (Å, °)

**Table:**

Cl1—C1	1.736 (3)	C3—C4	1.400 (4)
S1—C9	1.750 (3)	C3—H3C	0.9300
S1—C11	1.788 (4)	C4—C5	1.387 (4)
N1—C10	1.347 (3)	C4—C7	1.490 (4)
N1—C7	1.354 (3)	C5—C6	1.384 (4)
N2—C9	1.339 (3)	C5—H5A	0.9300
N2—C10	1.353 (4)	C6—H6A	0.9300
N3—C10	1.338 (4)	C7—C8	1.370 (4)
N3—H3A	0.8600	C8—C9	1.395 (4)
N3—H3B	0.8600	C8—H8A	0.9300
C1—C2	1.371 (5)	C11—H11A	0.9600
C1—C6	1.386 (4)	C11—H11B	0.9600
C2—C3	1.383 (4)	C11—H11C	0.9600
C2—H2B	0.9300		
			
C9—S1—C11	103.63 (16)	C5—C6—C1	118.7 (3)
C10—N1—C7	116.4 (2)	C5—C6—H6A	120.6
C9—N2—C10	115.1 (2)	C1—C6—H6A	120.6
C10—N3—H3A	120.0	N1—C7—C8	121.1 (3)
C10—N3—H3B	120.0	N1—C7—C4	115.6 (2)
H3A—N3—H3B	120.0	C8—C7—C4	123.3 (3)
C2—C1—C6	120.8 (3)	C7—C8—C9	118.2 (3)
C2—C1—Cl1	119.6 (3)	C7—C8—H8A	120.9
C6—C1—Cl1	119.7 (3)	C9—C8—H8A	120.9
C1—C2—C3	120.0 (3)	N2—C9—C8	122.4 (3)
C1—C2—H2B	120.0	N2—C9—S1	119.9 (2)
C3—C2—H2B	120.0	C8—C9—S1	117.7 (2)
C2—C3—C4	120.8 (3)	N3—C10—N1	117.1 (3)
C2—C3—H3C	119.6	N3—C10—N2	116.2 (2)
C4—C3—H3C	119.6	N1—C10—N2	126.7 (3)
C5—C4—C3	117.7 (3)	S1—C11—H11A	109.5
C5—C4—C7	120.8 (3)	S1—C11—H11B	109.5
C3—C4—C7	121.4 (3)	H11A—C11—H11B	109.5
C6—C5—C4	122.0 (3)	S1—C11—H11C	109.5
C6—C5—H5A	119.0	H11A—C11—H11C	109.5
C4—C5—H5A	119.0	H11B—C11—H11C	109.5

### Hydrogen-bond geometry (Å, °)

**Table:**

*D*—H···*A*	*D*—H	H···*A*	*D*···*A*	*D*—H···*A*
N3—H3A···N2^i^	0.86	2.31	3.095 (3)	152
N3—H3B···N1^ii^	0.86	2.21	3.045 (3)	164
C11—H11A···S1^iii^	0.96	2.93	3.859 (4)	163

Symmetry codes: (i) *x*+1/2, −*y*+5/2, −*z*+2; (ii) *x*−1/2, −*y*+5/2, −*z*+2; (iii) *x*−1/2, −*y*+3/2, −*z*+2.
